# The Exact Timing of Microinjection of Parthenogenetic Silkworm Embryos Is Crucial for Their Successful Transgenesis

**DOI:** 10.3389/fphys.2022.822900

**Published:** 2022-03-25

**Authors:** Valeriya Zabelina, Marketa Vrchotova, Naoyuki Yonemura, Hideki Sezutsu, Toshiki Tamura, Vyacheslav Klymenko, Frantisek Sehnal, Michal Zurovec, Hana Sehadova, Ivo Sauman

**Affiliations:** ^1^Biology Center CAS, Institute of Entomology, České Budějovice, Czechia; ^2^Faculty of Science, University of South Bohemia, České Budějovice, Czechia; ^3^National Agriculture and Food Research Organization, Tsukuba, Japan; ^4^Silk Sciences and Technology Research Institute, Ibaraki, Japan; ^5^Faculty of Automation and Information Technology in Management, Ryazan State Radio Engineering University, Ryazan, Russia

**Keywords:** transgenesis, parthenogenesis, *Bombyx mori*, embryonic development, genetic engineering, ovary transplantation, overcoming diapause

## Abstract

The use of parthenogenetic silkworm (*Bombyx mori*) strains, which eliminate the problem of recombination, is a useful tool for maintaining transgenic clonal lines. The generation of genetically identical individuals is becoming an important tool in genetic engineering, allowing replication of an existing advantageous trait combination without the mixing that occurs during sexual reproduction. Thus, an animal with a particular genetic modification, such as the ability to produce transgenic proteins, can reproduce more rapidly than by natural mating. One obstacle to the widespread use of parthenogenesis in silkworm genetic engineering is the relatively low efficiency of downstream transgenesis techniques. In this work, we seek to optimize the use of transgenesis in conjunction with the production of parthenogenetic individuals. We found that a very important parameter for the introduction of foreign genes into a parthenogenetic strain is the precise timing of embryo microinjection. Our modification of the original method increased the efficiency of transgene injection as well as the survival rate of injected embryos. We also provide a detailed description of the methodological procedure including a graphical overview of the entire protocol.

## Introduction

Transgene insertion is the critical step in the production of transgenic silkworms, but widespread use of this technique is hampered by the tedious selection and maintenance of desired genotypes. However, these procedures could be simplified by the use of parthenoclones. The main advantage of using parthenogenetic strains is that after successful integration of the transgene into the silkworm genome, a single female initiates a clonal lineage with a genome completely identical to that of the mother, which can be easily maintained as pure female populations without sexual reproduction. Subsequent generations have the same expression of the inserted gene and the same morphological and physiological characteristics. This is important for the production of proteins used in medicine, where it is necessary to ensure the same quality and quantity of the desired product (Tomita, 2011; Tatemastu et al., 2012), leading to the standardization of biotechnological and pharmacological sericulture products.

Parthenogenetic reproduction is induced in susceptible strains of *Bombyx mori* by specific thermal treatment of the unfertilized eggs of virgin females (Astaurov, 1940). The treatment, which involves heat shock followed by rapid cooling, suppresses the first meiotic division and both pronuclei of oocyte I remain diploid. One of them is aborted and the other becomes the nucleus of the oocyte II and initiates division (Klymenko, 2001). Since no crossing over of chromosomes occurs during silkworm oogenesis (Sturtevant, 1915), the pronuclei are genetically identical to the genome of the mother, including the ZW chromosomes that determine female sex (Tazima, 1978; Doroshenko and Klymenko, 2010). A single female initiates a female clonal lineage with the same genetic and morphological traits that are maintained without sexual reproduction (Tazima, 1978). Certain genotypes have been maintained as parthenoclones without sexual reproduction for decades (Astaurov, 1973; Klymenko et al., 2013; Zabelina et al., 2015b).

Considering that all available parthenogenetic strains of *B. mori* are diapausing, a way had to be found to obtain non-diapausing eggs for microinjection of foreign DNA. Therefore, the first successful attempt was to transplant ovaries into the male larval host (Zabelina and Klymenko, 2008; Zabelina et al., 2015a). Because males do not contain sufficient titers of the diapause hormone, eggs in ovaries implanted into male larvae lost embryonic diapause determination (Zabelina et al., 2015a). Recent results also suggest the possibility of overcoming diapause by crossing a diapausing parthenogenetic strain with a bivoltine strain (Zabelina et al., 2021).

The advantage of parthenocloning in the production of homozygous transgenic lines was first recognized by Grenier et al. (2004), who attempted to develop a parthenogenetic polyvoltine strain but failed to use the strain for transgenesis. The first parthenogenetic and simultaneously transgenic strains were constructed 10 years later but resulting efficiency of hatchability was less than 2% (Zabelina et al., 2015b). The parthenogenetic strain was incubated at 15°C and the DNA for the transgenes was injected 12 h after egg activation (AEA) by heat shock. The timing of the developmental stage for injection of developing embryo in 15°C was roughly estimated with respect to the fact that eggs incubated at 15°C developed approximately three times slower than at 25°C, where the optimal stage for DNA injection is determined to 2–6 h AEA for non-parthenogenetic strains (Tamura et al., 2000, 2007). At this stage of development, the energids divide synchronously, migrate to the periphery, and occupy approximately 25% of the anterior-lateral egg surface, where they begin cellularization. We hypothesized that the low efficiency of transgenesis in the parthenogenetic strain could be caused by incorrect estimation of the progress of the embryonic development at different incubation temperatures (15 vs. 25°C).

In this study, we compared in detail the course of early embryogenesis in parthenogenetic strain PK1 with European silkworm strain K23 at two different temperatures. We found out that the rate of early embryogenesis at 15°C is significantly slower than estimated in the previous study (Zabelina et al., 2015b). We have shown that accurate determination of the most appropriate developmental window for transgenic injection of parthenogenetic silkworm strains dramatically increased embryo survival.

## Materials and Methods

### Silkworm Cultures

Silkworm strain K23 (also known as Soviet-5) was used to study embryonic development after normal fertilization. The strain is univoltine, sex-marked in the egg stage and has a very low capacity for parthenogenesis (0.1%) (Strunnikov, 1971; Zabelina and Klymenko, 2008). The univoltine parthenoclone PK1 [also known as P29 (Astaurov, 1973], has been used to study embryonic development after induction of parthenogenesis by heat shock. The PK1 clone is characterized by high viability, the presence of multiple genetic markers, and nearly 100% parthenogenesis in heat shock treated eggs. Strains K23 and PK1 were kindly provided to us by the Agricultural Research Council (Padua, Italy). The non-diapausing parthenogenetic strain 1–31, the F2 brood between the PK1 and bivoltine Cambodia strains (Zabelina et al., 2021), was used for egg microinjection. All silkworms received artificial diet (Cappellozza et al., 2005) and were maintained under standard conditions of 15 or 25°C and a 12:12 h photoperiod.

### Transplantation of Ovaries

**FIGURE 1:**
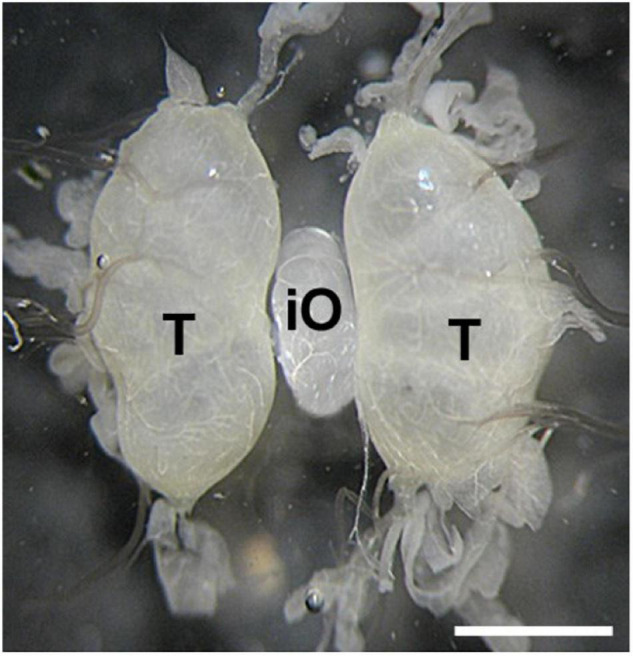
An examples of ovary implantation. Implanted PK1 ovaries (iO) positioned between the endogenous testes of the K23 recipient (T). Scale bar: 500 μm.

Transplantation of developing ovaries was used to interrupt diapause programming (Spiridonova et al., 1987). Day 4 PK1 larvae of the 4th instar were used as ovarian donors. Ovaries were dissected from water-anesthetized female larvae (20 min at room temperature) from the dorsal part of the 5th abdominal segment under sterile Ringer’s solution. The dissected ovaries were inserted into anesthetized recipient, day 3 male K23 larvae of the 4th instar, through a small incision on the dorsal side of the larva between the 5th and 6th abdominal segments. The donor ovary was carefully placed between the host testes using forceps and a needle. The site of surgery was sterilized with ethanol. Since males do not contain sufficient titers of the diapause hormone, the eggs in the implanted ovaries were not determined for embryonic diapause (Zabelina et al., 2015a). The transplanted ovaries were left in males throughout oogenesis. An example of ovarian implantation is shown in Figure 1.

### The Collection and Handling of Eggs

Eggs of the standard univoltine strain K23 were obtained as described by Tamura et al. (1990). Newly eclosed adults were mated at 25°C for 3–4 h and then left overnight at 5°C. Males were removed, and females were placed in darkness at 25°C, where they began laying eggs. Newly laid eggs were collected every 15–30 min, transferred to 15 or 25°C, and examined at 3 or 12 h intervals. Sperm penetration into the eggs during their passage through the oviduct triggered embryogenesis (Tazima, 1978; Miya, 2003) and the progress of embryonic development was conveniently measured in hours after egg laying. Hydrochloric acid (HCl) treatment (Astaurov, 1973) was used to break the diapause of K23 eggs incubated at 25°C.

Embryogenesis of unfertilized eggs of parthenogenetic strain PK1 was activated by heat shock treatment (Astaurov, 1940). Transplanted ovaries with fully developed eggs were dissected from newly eclosed adult K23 recipient males. The incision between the thorax and abdomen was made with fine scissors. The ovaries were removed and placed in a beaker of cold water. The dissected ovaries were scrubbed with circular movements of the fingers on a plastic sieve under running water to remove adjacent tissue. After another wash in cold water, the eggs were removed from the sieve, transferred to a gauze square, and placed in a small bag with a rubber band. The ovaries of the control group were collected from adult PK1 females after egg deposition and also placed in a gauze bag. Both groups of collected eggs were then heat shocked in warm water (46°C) for 18 min. To allow the bags to be immersed in the water, the air should be removed from the bags. The bags were then rapidly cooled by immersing them in 15°C water for 10 min. The bags were then dried with a towel and placed in the incubator at a temperature of 15°C and high humidity. The time of egg deposition for non-parthenogenic strains or heat shock activation of eggs of parthenogenic strains is defined as time zero after egg activation (AEA).

To determine the exact timing for injection of transgene DNA into the parthenogenetic strain PK1, we compared the embryogenesis rate of this parthenogenetic strain at 15°C with the univoltine strain K23 incubated at 25°C. The process of maintaining parthenogenetic individuals is based on eggs derived from the ovaries of the parthenogenetic strain PK1 implanted and grown in males of the strain K23, which prevents diapause due to the low content of diapause hormones in these males.

Embryonic developmental stages were determined based on the number and distribution of nuclei stained with propidium iodide and the degree of serosa formation. Based on the analysis of our samples and data published by Ohtsuki and Murakami (1968), Nagy et al. (1994), and Miya (2003), we distinguished 7 stages of early embryogenesis, designated A to G (Table 1). From this, we can conclude that in embryos maintained at 15°C, development slows down rapidly in both implants and PK1 and K23 strains.

**TABLE 1:** Brief description of the stages of early embryogenesis in *B. mori*.

Stage	Characteristics	Hours AEA		
		N25	O25	O15
A	One nucleus in parthenogenetic eggs or 2 pronuclei in fertilized eggs can be detected in exceptional cases.	0	0	0
B	Up to 40 nuclei assembled in the anterior third of the egg, nuclei are widely separated and some divide within the yolk.	7	6–12	12–24
C	Nuclei occupy about half of the anterio-lateral egg surface where they migrate to the periphery and initiate cellularization.	11	6–12	24–36
D	Nuclei occupy entire egg surface and form cellular blastoderm that contains (1) small cells of presumptive germ anlage in the lateral and ventral egg surfaces; (2) Larger preserosa cells peripheral to the germ anlage; (3) primary yolk nuclei in egg interior.	14	12–24	36–48
E	Large gaps between serosa cells, the cells of future germ anlage become more densely packed and begin to sink.	23	24–36	48–60
F	The rudimental anlage is clearly distinguished from the extraembryonic cells. The serosa is nearly continuous.	26	24–36	>72
G	The short anlage is enveloped by serosa and is separated from the extraembryonic cells.	NA	48–60	>72

*Hours indicate the approximate time of incubation after egg activation (AEA) when stages were reached in eggs incubated at 25°C (column N25 based on data published by Nagy et al. (1994) and column O25 based on our data) and in eggs incubated at 15°C (column O15).*

### The Fixation and Staining of Embryos

To remove the chorion and vitelline membrane, which interfere with embryo fixation and staining, eggs selected for cytology were placed in 30% KOH for 6 min followed by 2% NaClO for 3 min (Nagy et al., 1994; You et al., 2013). After washing in PBS (0.1 M, pH 7.4), the eggs were transferred to a fixative consisting of 2.5 ml PBS, 0.5 ml formaldehyde (38%) and 3 ml n-heptane. The samples were shaken for 50 min at 28°C. The fixative formed two liquid phases, the upper of which was discarded. The remaining liquid was carefully replaced with cold methanol in which the eggs were gently shaken for 10 min. The excess solution was removed and briefly washed three times with chilled methanol. The eggs were kept in fresh methanol and stored at −20°C. Prior to staining, samples were rehydrated through decreasing methanol series (90%, 70%, 50% and 30% for 15 min each) to PBS.

The cell nuclei were stained with propidium iodide (Sigma, 40 μg/ml), for 15 min in the dark (Nagy et al., 1994). Stained samples were washed three times in PBS for 15 min. Stained embryos were embedded in Vectashield medium (Vector Laboratories) on microscopic slides and stored in the dark at 4°C until examination with a confocal laser scanning microscope (FluoView™ FV1000, Olympus). Multiple Z-stacks images were combined using Imaris software (Bitplane, Oxford Instruments, plc, Tubney Woods, Abingdon, Oxon OX13 5QX, United Kingdom).

### Microinjection of Parthenogenetic Eggs

The successful transgenesis of parthenogenetic eggs was reported by Zabelina et al. (2015b) and improved in the following study (Zabelina et al., 2021). The activated eggs of non-diapausing parthenogenetic strain 1–31 were injected with the plasmid vector pPIGA3GFP carrying the GFP gene construct in order to confirm DNA injection (Tamura et al., 2000).

Preparation of plasmid DNA for injection: The plasmid DNA was purified by CsCl gradient ultracentrifugation. The recovered DNA was treated with 70% ethanol, dried by vacuum centrifugation, and dissolved in the injection buffer (5 mM KCl/0.5 mM phosphate buffer at pH 7.0) at a concentration of 200 μg/ml. The prepared DNA solution was stored at −80°C until injection.

Injection of DNA into developing embryos: The injection system consisted of a binocular microscope (Nikon, SMZ 745, Japan) with a stage, an injector (Narishige, IMP300, Japan), a manipulator (Narishige, M152, Japan), and a pipette holder (Narishige, HDD-20, Japan) (Tamura et al., 2007). First, the eggs were fixed on a slide in the same orientation and a small hole was pierced in the ventral side of the egg with a tungsten needle. The tip of a glass capillary was then guided to the location of the hole. By moving the knob on the movable stage of the microscope, the glass capillary could enter the egg. The DNA was then injected using the air pressure of the injector. Activated eggs developing at 15°C were injected with 1–4 nl of 200 μg/ml DNA within 72 h AEA at time points of 12, 18, 24, 30, 48, and 72 h. After injection, the developing embryos were brought to 25°C until hatching. After an additional 4 days of incubation at 25°C, the injected embryos were examined under an Olympus BX51 fluorescence microscope with CCD camera (Olympus DP80, Olympus Corporation, Tokyo, Japan).

Establishing *B. mori* transgenic lines: Parthenogenetic larvae hatched from transgene-injected eggs were reared to imago. Mature eggs were excised from the abdomen, heat shock treated, and examined under a fluorescence microscope for expression of the GFP marker. Transgenic strains were established from the GFP-positive embryos.

## Results

### Determining Optimal Staging for Transgenesis in the Parthenogenetic Silkworm Strain PK1

**FIGURE 2:**
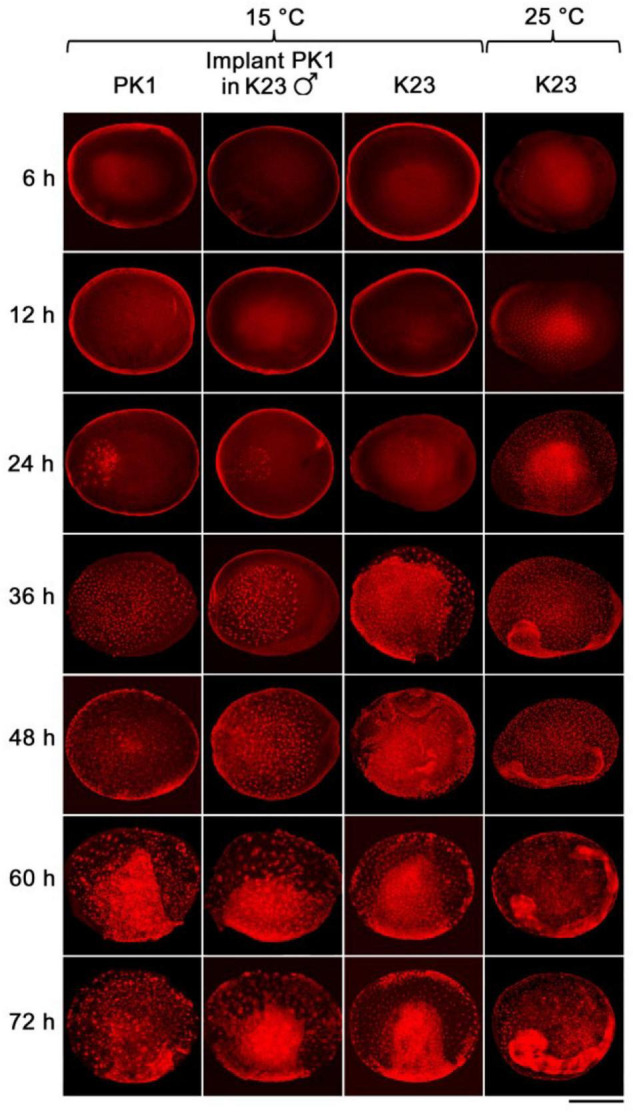
The rate of development of parthenogenetic and univoltine embryos of *B. mori* at different incubation temperatures. Embryogenesis was studied during 72 h AEA. Eggs were incubated at 15°C, except for the last column where embryos were maintained at 25°C. The time after egg activation is indicated on the left side of the panel. PK1, parthenogenetic eggs from PK1 ovaries; implant PK1 in K23 ♂, parthenogenetic PK1 eggs from ovarian implants that developed in K23 males; K23: fertilized eggs from endogenous K23 ovaries. Scale bar: 500 μm.

We examined the progress of early embryogenesis at 12-h intervals until 72 h after egg activation (AEA, except for the first time point at 6 h AEA) by propidium iodide staining and microscopic analysis (Figure 2). Later developmental stages were not examined in this study. Time point zero, i.e., egg activation, is activation of the parthenogenic strains by heat shock treatment or egg deposition in the non-parthenogenic strains. Embryogenesis of the implants was also compared with the development of the parthenogenetic eggs of PK1 left *in situ* in the intact ovaries and with the development of the standard fertilized eggs of K23, both incubated at 15°C (Figure 2).

As can be seen from the Figure 2, during the first 12 h AEA we did not observe pronuclei at 15°C. Thus, the first nuclei appeared between 12 and 24 h AEA. At the 24 h AEA stage, approximately one-third of the embryo is filled with dividing nuclei. In contrast, K23 embryos kept at 25°C already contain tents of dividing nuclei at 6 h AEA. At the 12 h AEA stage, the embryo is almost completely filled with nuclei. The difference in the rate of embryogenesis between 25 and 15°C increases further at later stages. Embryos developing at 25°C separate distinctly from the serosa between 24 and 36 h, whereas at 15°C the primitive germ band forms at 48 h and the anlage separates distinctly from the serosa at 72 h AEA. Individual strains show individual differences during incubation at 15°C, which become evident at 24 h AEA (Table 2). While in K23 about 40% of the tested embryos are at the stage of up to 40 nuclei, which is typical for the other two groups tested, in 60% of the embryos the nuclei occupy about half of the anteriolateral egg surface, migrate to the periphery and start cellularization there. In the implants, on the other hand, development is slightly delayed in the sense that from 36 h AEA onward, a higher percentage of less developed than advanced stages is found in the tested samples. The results of embryonic development at 15°C are summarized in Table 2.

**TABLE 2:** Stages of early embryogenesis in *B. mori* embryos incubated at 15°C.

Timing after egg activation (at 15°C)	PK1			Implant PK1 in K23 ♂			K23		
	Stage	%	*n*	Stage	%	*n*	Stage	%	*n*
12 h	A	100	12	A	100	16	A	100	11
24 h	B	100	16	B	100	14	B-C	40–60	12
36 h	C-D	40–60	10	C-D	75–25	15	D-E	80–20	10
48 h	D-E	10–90	11	D-E	65–35	16	E-F	80–20	27
60 h	F	100	14	F	100	2	F	100	12
72 h	F	100	10	F	100	9	F	100	21

*Embryonic development was analyzed at 12-h intervals during the first 72 h AEA. Each time point is characterized by developmental stages A-G described in Table 1. PK1, parthenogenetic eggs from PK1 ovaries; implant PK1 in K23 ♂, parthenogenetic PK1 eggs from ovarian implants that developed in K23 males; K23, fertilized eggs from endogenous K23 ovaries; n, number of embryos examined; %, percentage of occurrence of the embryonic developmental stage examined among all eggs in the sample. Stages of embryonic development were determined subjectively by two observers.*

**FIGURE 3:**
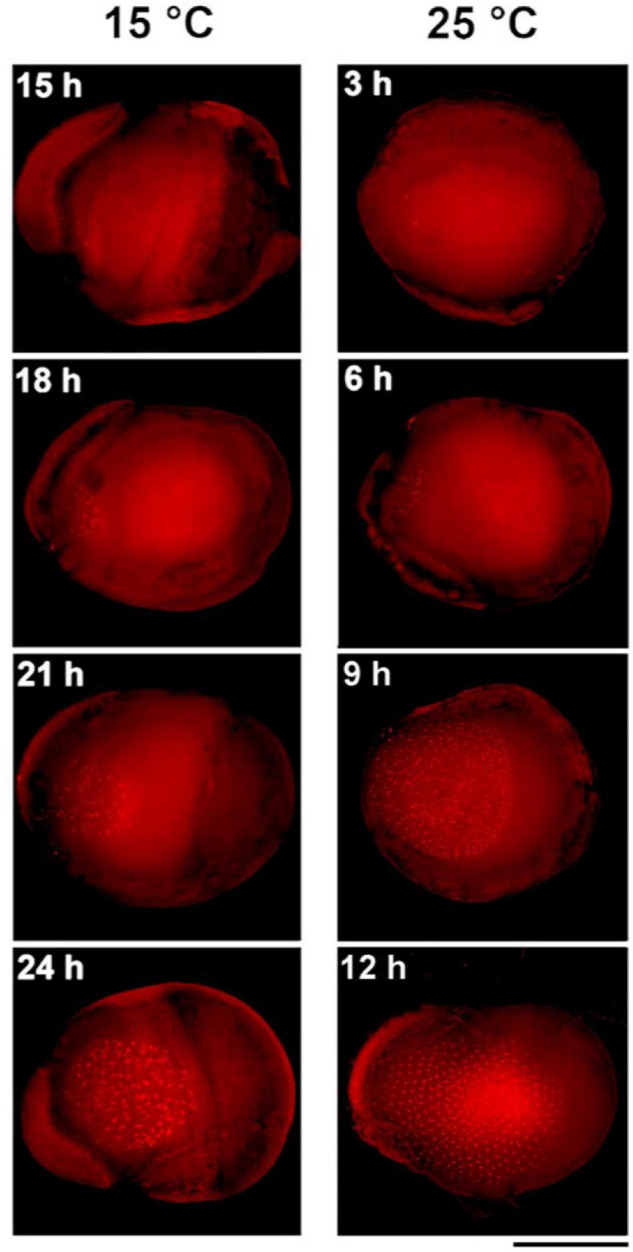
Comparison of early embryogenesis in standard breeding strain K23 incubated at 15°C and 25°C, respectively. The inserted numbers indicate the time after egg activation. Scale bar: 500 μm.

To obtain a more detailed picture of the earliest stages of embryogenesis, we examined the development of standard strain K23 every 3 h from 3 to 12 h AEA at 25°C and from 15 to 24 h AEA at 15°C (Figure 3). At 25°C, multiple nuclei were already detected at 3 h AEA, consistent with a previous observation that places the first division of the zygote to the time point between 120 and 140 min AEA (Tazima, 1978). At 15°C, the first nuclei were found at 15 h AEA. Based on these data, the most appropriate stage for DNA injection into parthenogenic embryos developing at 15°C is 18–24 h AEA. This stage corresponds to approximately 6 h AEA of embryos developing at 25°C.

### Efficiency of Microinjection at Different Stages of Early Embryogenesis

**FIGURE 4:**
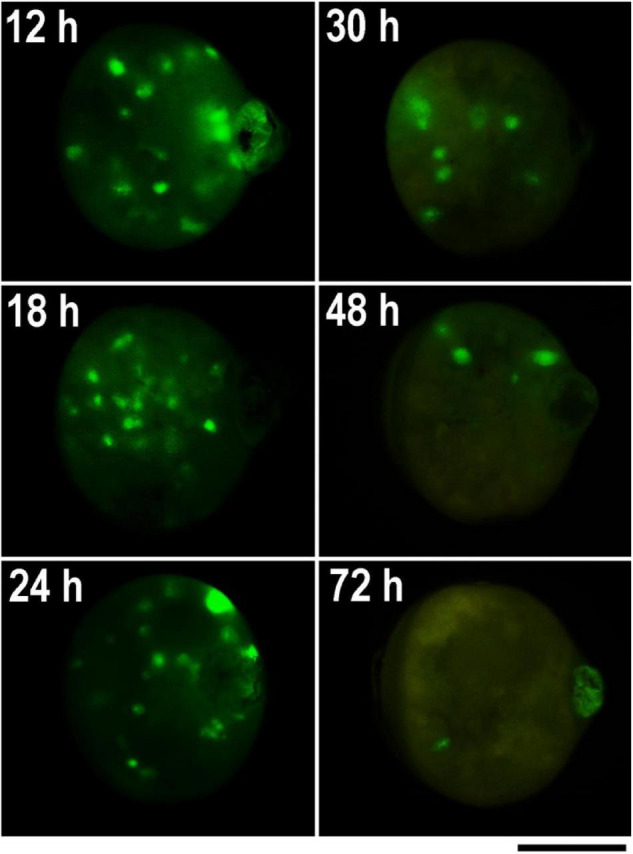
GFP expression in parthenogenetic embryos of *B. mori* 1–31 incubated at 15°C illustrating efficiency of microinjection at different developmental stages. The GFP-containing DNA plasmid was injected into the heat-shock-activated eggs during the first 72 h AEA in times specify by numbers in each figure. GFP expression was examined 4 days before hatching. Time AEA is indicated by the inset numbers. Scale bar: 500 μm.

**FIGURE 5:**
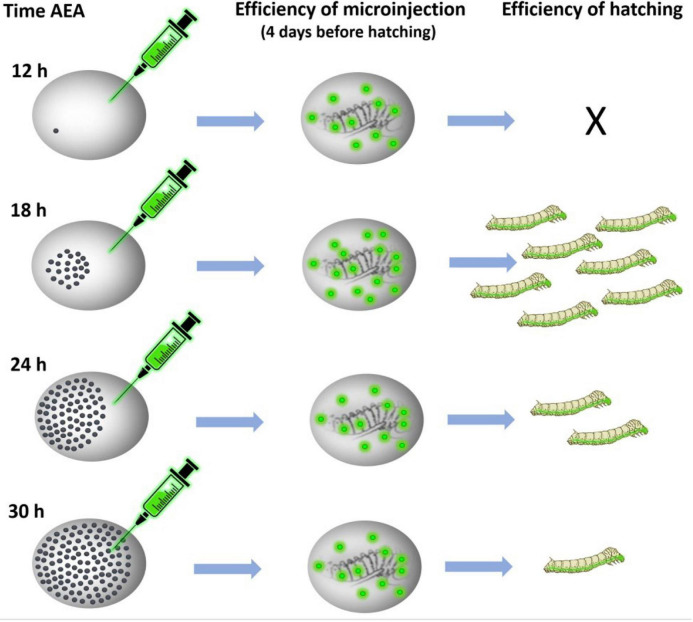
Schematic representation of the procedure of transgene microinjection. Time AEA, time after egg activation.

Considering the obtained results, we tested the embryo survival rate after DNA injection at different times AEA in the non-diapausing parthenogenetic *B. mori* 1–31 strain (Zabelina et al., 2021). To determine the most appropriate time point for transgenesis, the plasmid containing the GFP gene expression construct was injected into developing embryos that were activated by heat shock and maintained at 15°C for up to 72 h AEA. Then the embryos were maintained at 25°C until hatching. Four days later, the injected eggs were examined under a fluorescence microscope to determine the successful microinjection (GFP expression). We also assessed the efficiency of hatchability. The data are summarized in Table 3 and shown in Figures 4, 5. The highest efficiency of successful microinjection was observed in embryos injected between 12 and 30 h AEA. However, considering the hatchability of larvae after transgene injection, the most appropriate time for transgenesis was set at 18 h AEA (Table 3).

**TABLE 3:** Effect of embryonic stage on microinjection efficiency in embryos of parthenogenetic strain *B. mori* 1–31 incubated at 15°C.

Timing of injection after heat-shock activation (at 15°C)	No. of injected eggs	No. of died eggs	Expression of EGFP		Hatched larvae
			Not expressed	Expressed	
12 h	48	12 (25%)	2 (6%)	34 (94%)	0 (0%)
18 h	48	5 (10%)	3 (7%)	40 (93%)	7 (16%)
24 h	48	10 (21%)	2 (5%)	36 (95%)	2 (5%)
30 h	48	12 (25%)	1 (3%)	35 (97%)	1 (3%)
48 h	48	3 (6%)	30 (67%)	15 (33%)	3 (7%)
72 h	47	1 (2%)	33 (72%)	13 (28%)	16 (35%)

*GFP-containing DNA plasmid was injected into the heat-shock-activated eggs during the first 72 h AEA at the times indicated in the first column. The number of GFP-positive embryos examined 4 days before hatching reflects the efficiency of microinjection. Mortality of injected embryos and hatchability success of transgenic larvae were also examined. The corresponding percentages are given in parentheses.*

### Troubleshooting

To improve the survival rate of injected embryos, accurate determination of the optimal stage of embryonic development is critical. Assuming that the early embryonic development rate is similar among the different strains, we recommend the use of 18 h AEA stages. The rate of embryonic development in different strains has been compared in several studies to date. Early embryonic development of diapausing wild-type and non-diapausing *pnd* embryos maintained at 25°C showed no significant difference up to 36 h AEA (Sonobe et al., 1986; Nagy et al., 1994). Also, a comparison of the duration of total embryonic development in four different strains at five different temperatures ranging from 18 to 31°C showed no significant difference in the duration of embryonic development among the four strains (Mochida, 2001). However, in case of problems with survival of injected embryos, the rate of early embryogenesis of a particular strain at a particular temperature must be verified experimentally by morphological observations.

For accurate timing prior to actual transgene injection, we recommend using multiple biological replicates to determine the correct developmental window, especially when using different *B. mori* strains.

To visualize the dividing nuclei, we recommend staining with propidium iodide used in this study, which provides a stronger signal compared to staining with DAPI (Supplementary Figure 1). The crucial step for successful nuclear labeling is the precise removal of the chorion and vitelline membrane from the developing embryos.

### Graphic Summary of Protocol

(1A) Overcoming diapause by incubation in male.


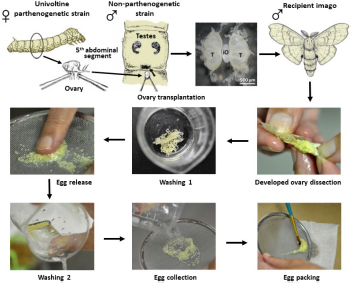


(1B) Overcoming diapause by crossing to bivoltine strain.


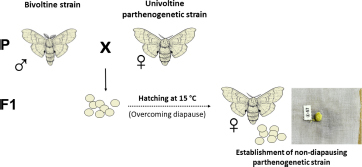


(2) Induction of parthenogenetic development.


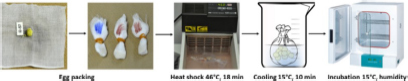


(3) Mechanism of ameiotic parthenogenesis.


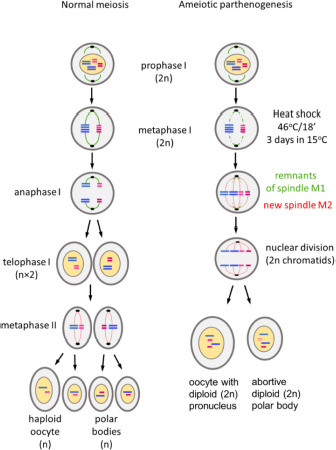


(4) Transgene injection at 18 h after heat shock.


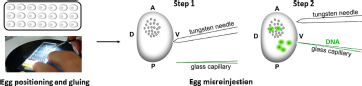


(5) Establishment of transgenic strains.


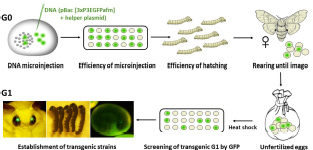


## Discussion

The introduction of key molecular biology techniques in silkworms opens the door to the genetic manipulations required for basic research and for the use of silkworms as tools for biotechnological production of recombinant proteins. Following the introduction of transgenesis and targeted mutagenesis methods, there is a need to increase the efficiency of transferring important traits and their combinations to progeny by producing genetically identical individuals. In this work, we focus on the optimal matching of the methods used for genetic modification with induced parthenogenesis, which allows the production of silkworm parthenoclones.

A common problem in genetic manipulations is the detection and maintenance of genetic changes in modified individuals, which depends largely on the laborious genotyping of offspring in each generation. Induced parthenogenesis, which allows the production of identical individuals, ensures the transmission of desired traits without the admixture that occurs during sexual reproduction (Grenier et al., 2004; Zabelina et al., 2015b,^2021^).

The technique of silkworm transgenesis is based on the injection of DNA constructs into the embryo at a very early stage of nuclear division, before the formation of the cellular blastoderm (Tamura et al., 2000, 2007; Uhlirova et al., 2002; Tomita et al., 2003; Inoue et al., 2005; Royer et al., 2005; Dai et al., 2007; Zhong et al., 2007; Zhao et al., 2010). The transgene, along with adjacent DNA, is randomly inserted into the genome of future somatic or germ cells. Transgenic cells (or individuals) can be detected by markers encoded in the construct.

Today, there is a number of techniques that can be used to edit the genome of various organisms, including insects. One of these is *piggyBac* technology (Tamura et al., 2000), which uses a transposon-derived vector discovered in the lepidopteran *Trichoplusia ni* (Cary et al., 1989) to introduce various genetic constructs (genes) into different insect species. However, the disadvantage of this method is that it does not allow precise targeting of the inserted genetic information. In contrast, endonuclease-based techniques such as zinc fingers, TALENs, and more recently CRISPR/Cas9 methods enable precise targeted genome editing, especially modification of selected genes. Targeted mutagenesis methods use essentially the same basic scheme of embryo microinjection and differ only in the quality and quantity of injected material and the detection of induced genetic changes (Takasu et al., 2010, 2016a,b). Both types of transgenic approaches have different applications in genetic engineering. Another option for transgenesis is homologous recombination, which has the advantage that whole genes or parts of them can be exchanged *in situ*. This method has been successful in *B. mori* using a baculovirus-derived vector, but with extremely low efficiency that precludes routine use (Yamao et al., 1999).

Success of transgenesis depends greatly on accurate staging of target embryos. Injection should be performed before blastoderm formation, i.e., no later than 12 h AEA at 25°C (Tazima, 1978). Tamura et al. (1990) have shown that injection of transgenes under standard conditions at 25°C is efficient during the first 8 h AEA, whereas injections 4 h later (after blastoderm formation) are ineffective. Standard strains developing at 25°C were therefore injected 2–6 h after oviposition (Tamura et al., 1990; Uhlirova et al., 2002), with a hatching rate of about 40% (Tamura et al., 2007). The parthenogenetic strain developing at 15°C was injected 12 h AEA, with a hatching rate of less than 2% (Zabelina et al., 2015b). Subsequent experiments with non-diapausing parthenoclones, where the developmental window for injection was extended from 12 to 18 h AEA, increased the percentage of hatchability by about 10-fold (Zabelina et al., 2021). Since our work shows that the embryonic stage corresponding to 2–6 h at 25°C was reached later during incubation at 15°C than the originally estimated 12 h after heat shock, between 18 and 24 h (Figures 2, 3 and Table 2), the likely reason for the low efficiency of hatchability in the previous study (Zabelina et al., 2015b) could be the inappropriate timing of DNA injection into the clonal strains.

We tested this assumption using the non-diapausing parthenogenetic line 1–31 (Zabelina et al., 2021) and showed that DNA injection at the correct developmental stage increased the percentage of hatching embryos from 0% at 12 h AEA to 16% at 18 h AEA (Figure 5). Thus, injection of DNA at the correct embryonic developmental stage significantly increases the hatchability of eggs and improves the efficiency of transgenic silkworm production as well as targeted mutagenesis. We believe that a detailed description of the whole procedure of parthenogenesis induction in connection with the production of transgenic silkworms will allow a wide application of this method in further research.

## Data Availability Statement

The original contributions presented in the study are included in the article/Supplementary Material, further inquiries can be directed to the corresponding author/s.

## Author Contributions

VZ, VK, FS, TT, HaS, and IS: conceptualization and study design. VZ, MV, NY, TT, and HaS: experiment performance. VZ, MV, TT, HaS, and IS: data analysis. FS, HaS, and TT: supervision. FS, MZ, HaS, and IS: writing—original draft. VZ, MV, NY, HaS, TT, VK, FS, MZ, HiS, and IS: manuscript revision and editing. All authors contributed to the article and approved the submitted version.

## Conflict of Interest

The authors declare that the research was conducted in the absence of any commercial or financial relationships that could be construed as a potential conflict of interest.

## Publisher’s Note

All claims expressed in this article are solely those of the authors and do not necessarily represent those of their affiliated organizations, or those of the publisher, the editors and the reviewers. Any product that may be evaluated in this article, or claim that may be made by its manufacturer, is not guaranteed or endorsed by the publisher.

## Supplementary Material

Supplementary Figure 1 The nuclei in embryos of parthenogenetic PK1 strain incubated at 15°C 24 h after egg activation stained by propidium iodide (red) and by DAPI (blue). Scale bars: 500 μm.
