# Supplementary figures and images for: The Exact Timing of Microinjection of Parthenogenetic Silkworm Embryos Is Crucial for Their Successful Transgenesis
Source: Front Physiol. 2022 Mar 25;13:822900. doi: 10.3389/fphys.2022.822900 (PMC8990321; doi:10.3389/fphys.2022.822900)

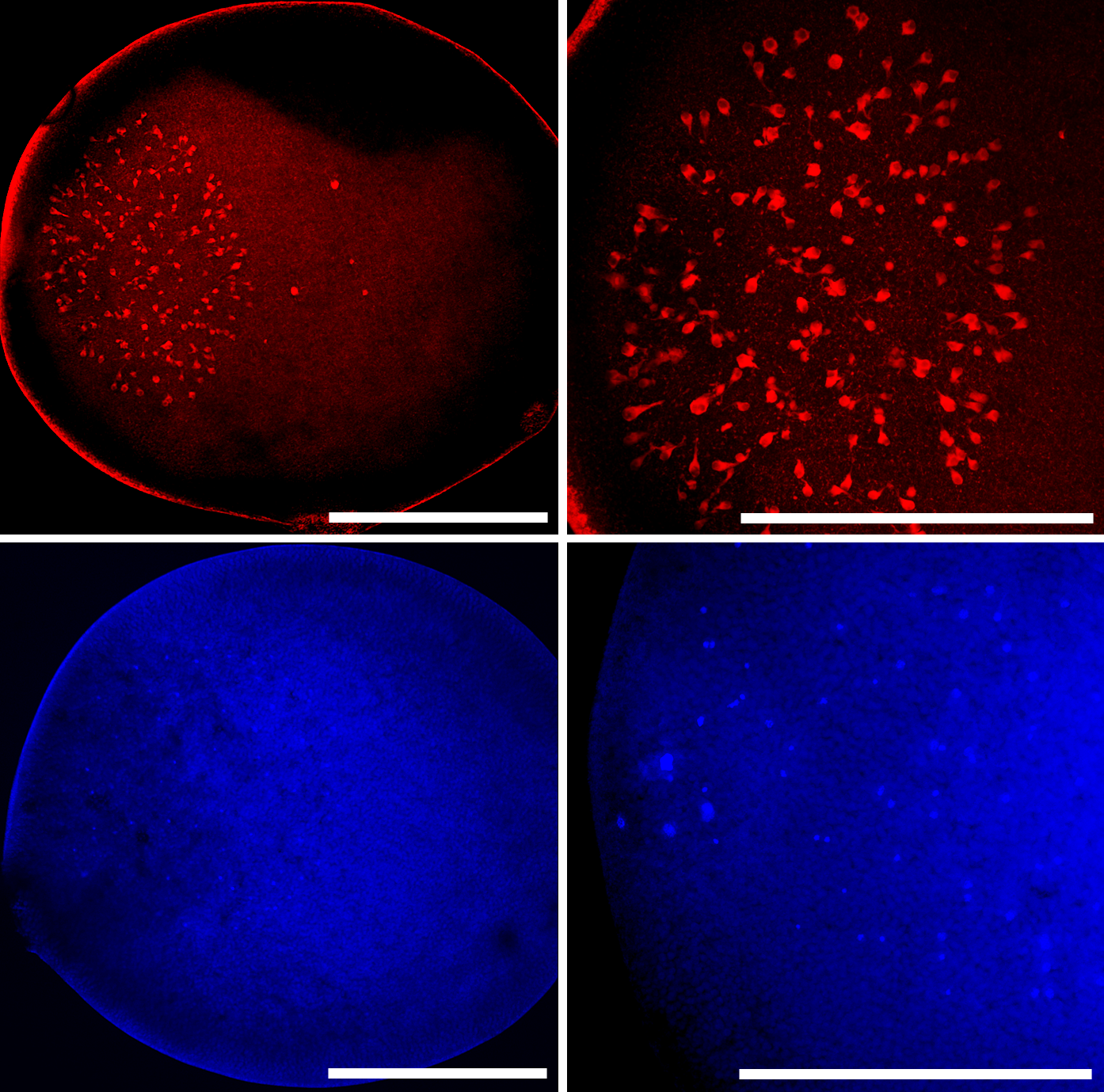

Supplement: Supplementary Figure 1 — The nuclei in embryos of parthenogenetic PK1 strain incubated at 15°C 24 h after egg activation stained by propidium iodide (red) and by DAPI (blue). Scale bars: 500 μm. [file Image_1.TIF]
